# Antibiotics Amoxicillin, Ampicillin and Their Mixture—Impact on Bacteria, Fungi, Ostracods and Plants

**DOI:** 10.3390/molecules29184301

**Published:** 2024-09-11

**Authors:** Barbara Pawłowska, Marcin Sysa, Agnieszka Godela, Robert Biczak

**Affiliations:** The Faculty of Science and Technology, Jan Długosz University in Czestochowa, 13/15 Armii Krajowej Av., 42-200 Czestochowa, Poland; m.sysa@ujd.edu.pl (M.S.); a.godela@ujd.edu.pl (A.G.); r.biczak@ujd.edu.pl (R.B.)

**Keywords:** antibiotics, *H. incongruens*, wheat, bacteria, fungi

## Abstract

Ampicillin (AMP) and amoxicillin (AMX) are popular antibiotics, which are penicillin derivatives, and are used in both human and veterinary medicine. In the conducted study, AMP, AMX and their mixtures did not cause major changes in the total bacterial counts in soil samples, and even an increase in the bacterial counts from 3,700,000 to 6,260,000 colony-forming units (cfu) per gram of soil dry weight (g of soil DW) was observed for minimal amounts of these drugs in the soil. The total abundance of fungi, on the other hand, increased from values ranging from 17,000 to 148,000 cfu∙g^−1^ of soil DW to a level of 32,000 to 131,000 cfu∙g^−1^ of soil DW. The tested antibiotics and their mixtures had no significant effect on the mortality and growth of *H. incongruens*. AMX and the AMP + AMX mixture also showed no effect on the plant fresh weight yield, plant aboveground part length and dry weight content of wheat seedlings. In contrast, AMP caused an increase in the plant fresh weight yield and wheat seedling length compared to the control. The drug also caused a slight decrease in the seedling dry weight content. Both AMP and AMX showed inhibitory effects on the plant root length at the highest concentrations of the compounds.

## 1. Introduction

Alexander Fleming’s discovery of the first antibiotic, penicillin G, in 1928, was a milestone in medicine, enabling doctors to fight many diseases. Since then, there have been enormous advances in the field and we now have a very large number of natural, semi-synthetic and synthetic antibiotics [1,2]. However, in addition to the undoubted benefits of the availability of antibiotics, there have also been drawbacks to their use. Numerous scientific reports indicate that antibiotics administered to humans and animals are not completely retained in the body. Many of the drugs we take are excreted from the body and end up in the environment [3,4]. Antibiotics given to animals are also excreted by them and end up on fields, from where they can be leached into waterways [5]. Wastewater treatment plants cannot cope with the removal of such pollutants, so these compounds continue to spread and have an increasing impact on ecosystems [3]. At the same time, antibiotics found in the soil, either directly from animals or as a result of fertilizing a field or irrigating with wastewater, can be taken up by plants or other organisms in the soil [5]. Although the adverse effects of the intake of antibiotics found in plants are not well understood and studied, these compounds can cause allergic or even toxic reactions in humans. In addition, antibiotics cause the development of multi-resistant bacteria, which are a major threat to human and animal health. At the same time, once in the environment, antibiotics can affect natural microbial communities that play a key role in maintaining soil and water quality, among other things. Available studies show that antibiotics can have a negative effect on the yield of plants grown on soils containing them. At the same time, their negative effects on photosynthesis and antioxidant metabolism limit the ability of plants to perform phytoremediation [2,6].

An additional complication is that antibiotics and their transformation products exhibit a wide range of physicochemical and biological properties depending on the abiotic properties of the environment. Under different pH conditions, they can behave as inert, cationic, anionic or hermaphroditic compounds, which may result in different toxicity or antibiotic reactivity [1]. It is important to remember that antibiotics do not exist as single compounds in the environment. The presence of different types of drugs in the environment, including antibiotics or other types of xenobiotics, can result in a synergistic effect. Antibiotics, like other drugs, are considered “pseudo-persistent” pollutants due to their continuous presence in the environment. Even though drugs decompose relatively quickly, they are still constantly present in the environment because we are constantly putting them there [1,7].

Penicillin and its derivatives are among the most widely used antibiotics in the world, both in human and veterinary medicine. Examples of this type of antibiotic are ampicillin and amoxicillin, which belong to the β-lactam antibiotics of the aminopenicillin group. They are semi-synthetic antibiotics with a broad spectrum of activity. They are effective against both Gram-positive and Gram-negative bacteria, but ampicillin is much less effective against Gram-positive bacteria than against Gram-negative bacteria [3,8]. As mentioned above, most drugs are eliminated from the body unchanged or as various types of metabolites. About 80% of amoxicillin is eliminated from the body. In addition, a study by Alexy et al. [9] shows that this antibiotic is not degraded in surface water and wastewater treatment plants.

Almost 70 years ago, Rosen discovered that antibiotics such as streptomycin could have an effect on plants [10]. Xie et al. [11] and Migliore et al. [12] also point to a hormesis effect in plants following exposure to low concentrations of antibiotics. Despite ongoing research, little is known about the effects of individual antibiotics and their mixtures on the environment, including plants. However, if antibiotics have any effect on plants, i.e., if we eat such plants and their crops or the animals that have fed on them, we also ingest the compounds they have absorbed in minimal amounts. Therefore, this paper presents a study to determine the effects of ampicillin and amoxicillin and their mixture on the growth and development of one of the world’s most popular cereals—wheat. In addition, to provide a more complete picture of the effects of these compounds on the environment, their effects on the crustacean *H. incongruens*, bacteria and fungi were determined. In this article, the total abundance of culturable bacteria and fungi was determined and extended to include the isolation of bacteria of the genus *Pseudomonas* and *Staphylococcus*, yeasts and filamentous fungi, which were most abundant in the soil used for the study. The isolated microorganisms belong to the autochthonous flora of the ecological niche from which the substrate used for the study originated. Throughout, the research was carried out on the natural microflora of the soil and no allochthonous strains were introduced into the soil.

Many fields are located close to bodies of water. The two ecosystems, aquatic and terrestrial, therefore interact closely. Benthic organisms, which are in contact with both water and sediment, are particularly vulnerable to the associated pollution. Such organisms include the crustacean *H. incongruens*, which is also very sensitive to any pollution in its habitat. In this way, we can learn the answer to the question of how antibiotics present in and leached from the soil affect benthic organisms.

## 2. Results and Discussion

### 2.1. Bacteria and Fungi

**Figure 1:**
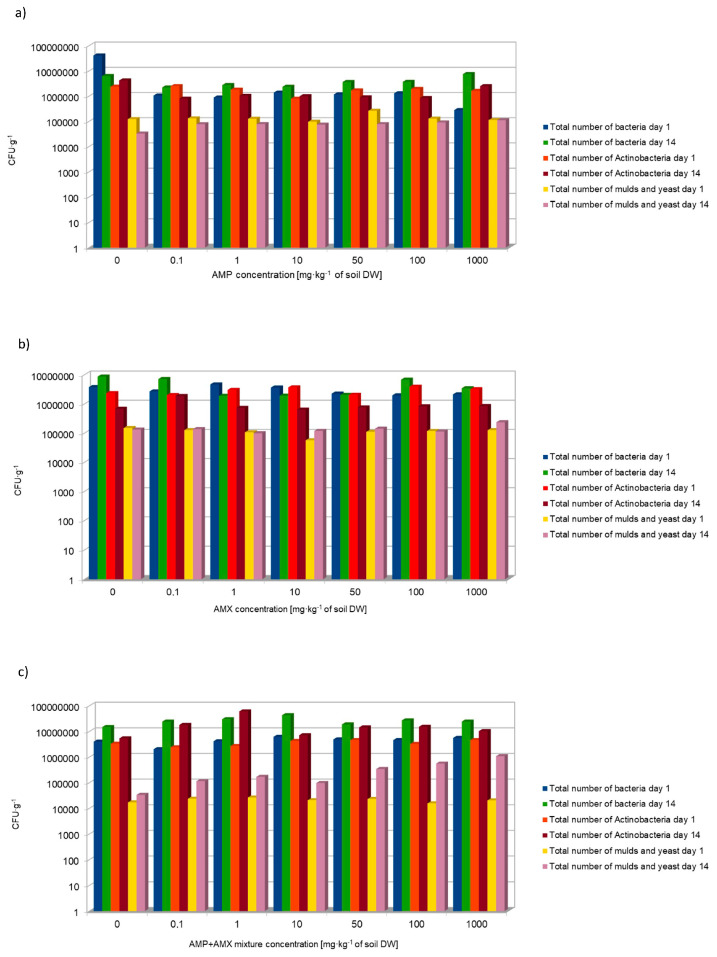
Effect of AMP (**a**), AMX (**b**) and AMP + AMX mixture (**c**) applied at concentrations of 0.1–1000 mg∙kg^−1^ of soil DW on total bacterial abundance in soil.

The results showed that the total abundance of bacteria in the soil samples of the control groups ranged from 3,700,000 to 40,800,000 colony-forming units (cfu) per gram of soil dry weight (DW) on the first day of the tests conducted. On the 14th day of the tests, the total bacterial abundance ranged from 6,260,000 to 8,610,000 cfu·g^−^^1^ DW (Figure 1).

The total number of fungi in the control groups on the first day of the tests ranged from 17,000 to 148,000 cfu·g^−^^1^ DW. On day 14, the values ranged from 32,000 to 131,000 cfu·g^−^^1^ DW. Such a large variation in the number of microorganisms in the soil, although it hinders the statistical development of the results and reference points, is a physiological response of the environment to physicochemical factors [13]. The extent to which bacteria and fungi populate the soil has been shown to be closely related to the amount of organic matter and the availability of oxygen [14].

**Figure 2:**
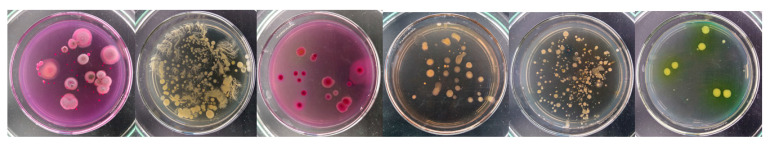
Plate cultures of individual groups of microorganisms in the control soil sample. From left: TSBA medium—total bacterial abundance; DRBC medium—total abundance of filamentous fungi and yeasts; VRBG medium—total abundance of *Enterobacteriaceae* family bacteria; MSA medium—total abundance of *Staphylococcus* family bacteria; AIA medium—total abundance of actinomycetes; PFA medium—total abundance of *Pseudomonas* family bacteria.

The aim of this study was to investigate the effect of increasing concentrations of ampicillin, amoxicillin and mixtures of these antibiotics on the total abundance of selected groups of soil microorganisms—*Pseudomonas*, *Enterobacteriaceae*, and *Staphylococcus* bacteria, filamentous fungi, yeasts and Actinomycetes (Figure 2). All of these groups of microorganisms are present in the soil and have a significant influence on both the circulation of matter and the fertility of the soil itself [15].

**Figure 3:**
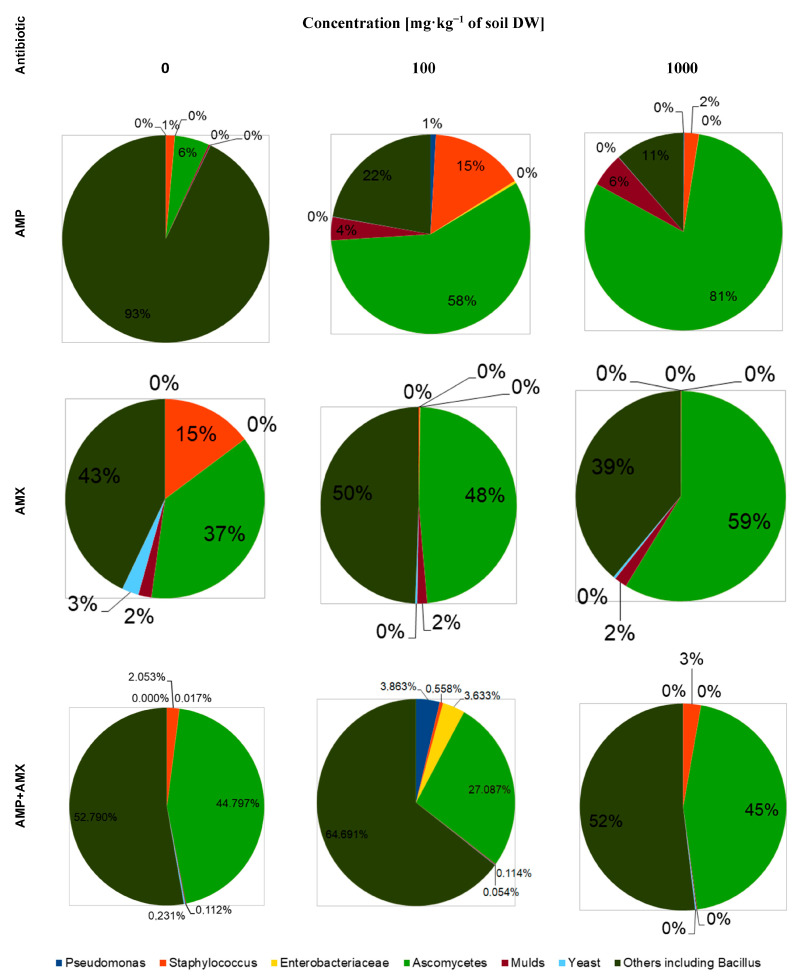
Effect of amoxicillin, ampicillin and their mixture at different concentrations on the percentage composition of soil microflora in soil samples taken on the day of plant culture establishment.

**Figure 4:**
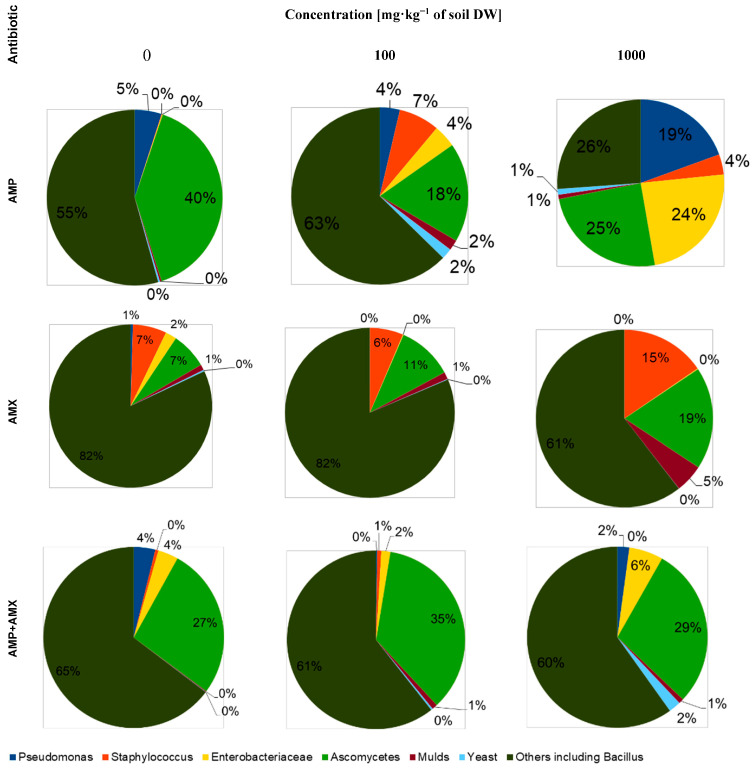
Effect of amoxicillin, ampicillin and their mixture at different concentrations on the percentage composition of the soil microflora in soil samples taken on the day of plant culture liquidation.

Numerous studies and literature reports indicate that both ampicillin and amoxicillin have a broad spectrum of activity against both Gram-positive and Gram-negative bacteria [16,17]. In our study, not only did increasing concentrations of these antibiotics not result in a decrease in the total abundance of microorganisms compared to the control group 14 days after the start of the experiment, but in the case of a mixture of these compounds, we observed an increase in the total abundance of both bacteria and fungi, regardless of the applied concentration of the tested drugs. The lack of a negative effect of the antibiotics on the total microbial content of the soil can be explained by the following: the ability of the microorganisms to biodegrade antibiotics, the decrease in their availability over time due to the aerobic degradation of the lactam ring, and the natural ability of microorganisms to generate resistance [16,18]. In addition, the intrinsic interaction between the two β-lactam antibiotics should be taken into account, which may result in the competitive inhibition of ampicillin by amoxicillin, but the mechanism of these relationships requires further research [19]. A slight decrease in the number of bacteria at the beginning of the experiment, independent of the dose of each antibiotic applied, can be explained by the death of the most sensitive groups of microorganisms [18]. The observed overall increase in the abundance of fungi in the tested soil seems interesting in this context. Nolan et al. [20] showed that the presence of ampicillin stimulates phosphorylation processes in fungal cells (especially in yeasts), resulting in an increase in their abundance [20,21,22]. In the studies carried out, we find confirmation of this phenomenon, since both an increase in the dose of ampicillin and the duration of exposure to it directly translate into a percentage of fungi in the total population of microorganisms present in the soil (Figure 3 and Figure 4).

For amoxicillin, on the other hand, we observed a comparable total fungal abundance, independent of dose and exposure time, confirming the conclusions of Spatz et al. [23], who showed an inhibitory effect of *Enterobacteriaceae* on fungal colony growth (Figure 3).

The mechanism of this relationship is not fully understood, but it seems to be strictly dependent on the presence of other groups of bacteria interacting with *Enterobacteriaceae* modulating, to different degrees, their effect on the development of yeast colonies. These interactions could explain why, in the groups where the antibiotic mixture was applied after 14 days of the experiment, we can observe an increase both in the number of Entebacteriaceae and in the percentage of fungi in the total population of the microorganisms. Correlating with the above statement is the study by Viçosa et al. [24], which showed that in an environment where bacteria of the *Staphylococcus* and *Enterobacteriaceae* families are present, the growth of the former is inhibited as a result of the population dominance of *Enterobacteriaceae*. Our own research presented in this paper seems to confirm this interaction, showing that regardless of the dose of antibiotics, their mixtures and exposure time, the overall abundance of *Staphylococcus* is reduced relative to the beginning of the experiment, while the abundance of *Enterobacteriaceae* is increased (Figure 3 and Figure 4).

The reduction in the number of *Staphylococcus* is a welcome development from an agricultural perspective, as this group of bacteria does not occur naturally in soil and is an indicator of soil contamination by substances of human or animal origin [25]. Similarly, the presence of *Enterobacteriaceae* is undesirable agriculturally, belonging to the group of so-called FIB (Fecal Indicator Bacteria)—bacteria that indicate that there has been water/soil contamination by fecal bacteria of human or animal origin [26]. The presence of *Pseudomonas* bacteria in soil has been confirmed by numerous scientific publications, but their effect on soil fertility, and thus on the physiological state of plants depends directly on the specific species [27]. Each *Pseudomonas* species isolated from soil/plants has shown a tendency to be a saprophyte or a leaf parasite. Some (such as *P. chlororaphis*) promote plant growth by inhibiting the proliferation of pathogens, stimulating the production of plant growth hormones or activating genes responsible for disease resistance, while others, under the right conditions, contribute to mold development (*P. fluorescens*) [28]. In the studies presented in this publication, we found no inhibitory effect on the growth of *Pseudomonas* after 14 days of the experiment, regardless of the type of antibiotic tested, their mixtures, concentration or exposure time. In addition to the mixture of antibiotics at a dose of 100 mg·kg^−^^1^ of soil DW, an increase in the percentage of *Pseudomonas* in the total population of microorganisms in the soil samples was observed after 14 days. The lack of inhibition by the tested antibiotics may be due to the previously mentioned ability of the bacteria to biodegrade and develop resistance or the insufficient exposure time [29]. Future research should be extended to verify the taxonomic affiliation of the bacteria in the *Pseudomonas* family and to test the efficacy of the antibiotics over a longer period of time.

### 2.2. Ostracodtoxkit

*Heterocypris incongruens* are microscopic-sized crustaceans found in waters around the world. Very important for their development is the fact that their eggs can survive dry periods as they are resistant to desiccation. Clams are sensitive to pollution in their habitat. Because they live in the bottom sediments of water bodies, they are sensitive to any contaminants present in both the water and the sediment [30,31].

As a result of the studies conducted on the effect of different concentrations of AMP, AMX and the AMP + AMX mixture, no effect of the tested antibiotics on the mortality of *H. incongruens* was observed. The tested antibiotics also did not cause inhibition of clamshell growth (Table 1).

The lack of an effect of hospital sludge containing different types of drugs on clamshells was reported by Perrodin et al. [32]. However, the antibiotic oxytetracycline and NSAIDs, i.e., ketoprofen, ibuprofen, naproxen or indomethacin, had a significant effect on *H. incongruens* mortality [33]. This means that the observed effect on ostracods is dependent on the type of drug used. However, due to the fact that there are very few studies available on the effects of drugs on this shellfish, and most of these studies are on the effects of NSAIDs, this makes it difficult to assess the real effects of drugs, including antibiotics, on benthic organisms such as *H. incongruens*. An additional complication is the fact that drugs do not occur in nature on their own, but in various types of mixtures with various other contaminants. Thus, although a given drug may not pose a threat to *H. incongruens* on its own, it may pose a major threat to benthic organisms in combination with another drug or other compound. In addition, the magnitude of the effect is strongly influenced by environmental conditions, which are constantly changing.

### 2.3. Phytotoxicity

An analysis of the results shows that both ampicillin (AMP) and amoxicillin (AMX) and their mixture, regardless of the concentration used, had no significant effect on the germination potential and germination capacity of the wheat seeds (Table 2).

AMX and the AMP + AMX mixture also had no effect on the plant fresh weight yield, aboveground plant length and dry weight content of wheat seedlings. In contrast, AMP increased the fresh weight yield and wheat seedling length compared to the control. It also caused a slight decrease in the dry weight content. Both AMP and AMX showed effects on the plant root length. AMX caused faster root growth when lower concentrations of the compound were applied, and a slight inhibition of root growth when a concentration of 1000 mg·kg^−^^1^ of soil DW was applied. AMP, on the other hand, caused a slight inhibition of root length at concentrations of 100 and 1000 mg·kg^−^^1^ of soil DW. The AMP + AMX mixture did not cause major, statistically significant changes in the wheat root length (Table 3).

A much more pronounced effect of antibiotics, i.e., AMP and AMX, on rice was observed by Mukhtar et al. [34]. In their study, the presence of antibiotics caused the inhibition of the length of the rice seedlings and their roots. Li et al. [5], in their study on the effects of oxytetracycline (OTC) and enrofloxacin (ENR) on wheat, indicate that antibiotics can affect seed germination in different ways, and none of the antibiotics tested by the cited authors had any effect on the number of germinated seeds. This may be because seed coats protect seeds by limiting the penetration of antibiotics into the seed. If this protective mechanism works efficiently, seeds sown in the soil are relatively resistant to many environmental stresses. However, this lasts only until the seed coat breaks down. OTC and ENR had no effect on the number of seeds that germinated, but OTC had a significant effect on the germination potential. At the same time, both antibiotics tested affected the length of the wheat seedlings and the number and length of the roots. Similar observations were made by Bellino et al. [35] in their study on the effects of chloramphenicol (CAP), spiramycin (SPR), spectinomycin (SPT) and vancomycin (VAN) on tomato. These authors also observed no effect of the tested antibiotics on seed germination.

Migliore et al. [12], studying the effects of enrofloxacin on cucumber, lettuce, radish and beans, observed the effects of this antibiotic on all plants. These authors found that the tested antibiotic caused hormesis in plants when it was applied at lower concentrations, whereas the inhibition of the plant and root growth was found when higher concentrations of the tested drug were applied. Meanwhile, Riaz et al. [36] investigated the effects of fluoroquinolone antibiotics on wheat. The study found that the antibiotics in this group had no effect on seed germination, but showed significant effects on plant growth and development. However, regardless of how the antibiotic affects plant growth, any changes clearly show that the drugs, but also their metabolites, are absorbed by plants and accumulate in various plant organs, affecting their growth and development.

However, the presence of drugs and their effects on plants is not always evident in the external appearance of the plant. Pierattini et al. [37], studying the effects of erythromycin on white poplar, did not observe any changes in the appearance of the tree or its roots. However, after careful analysis, erythromycin was detected in all the plant organs, with many times more erythromycin in the roots than in the other organs.

At the same time, it should be remembered that some drugs are very readily degraded, resulting in the formation of various types of metabolites, that may also have effects on living organisms including plants. Fu et al. [38], studying the effects of various antibiotics on the green alga *Pseudokirchneriella subcapitata*, showed that drugs such as amoxicillin can be easily photodegraded or hydrolyzed. Thus, the toxic effects of drugs can be caused not only by the drugs themselves, but also by their metabolites.

However, regardless of whether we are observing the effect of the drug itself, a mixture of drugs or their metabolites, the studies cited above indicate that the sensitivity of different plant species to antibiotics varies. The effect of drugs, including antibiotics, on plant growth is very complicated, depending on the plant species, the concentration and type of antibiotics, and the physiological stage of the plant organism.

## 3. Materials and Methods

### 3.1. Chemical Reagents

Ampicillin (anhydrous, 96.0–102.0%) and amoxicillin (potency: ≥900 μg per mg) were purchased from Sigma-Aldrich Chemical Co., (Poznan, Poland).

### 3.2. Determination of Total Bacterial and Fungal Counts

This study was carried out following the procedure of Ilesanmi et al. [39], extended to examine the qualitative composition of the microflora present in the soil samples. Soil samples were taken during the establishment of the plant culture and at the end of the culture after 14 days. The tests were performed immediately after soil sampling. Microorganisms were isolated by shaking a 10 g soil sample in 90 mL of 0.85% sterile sodium chloride (NaCl) solution for 15 min on a laboratory shaker at 150 rpm. Using the serial dilution method (1:10), such dilutions of the soil suspension were obtained that countable number of bacterial and fungal colonies were obtained after incubation. The soil suspension from each dilution was seeded using the surface seeding method onto sterile Petri dishes (sterilized in an autoclave at 121 °C, 1.3 atmospheres pressure for 15 min) containing specific media:

- Tryptic Soya Agar to determine total bacterial abundance (TSBA);
- Dichloran Rose Bengal Chloramphenicol (DRBC) to determine total mold and yeast abundance;
- Mannitol Salt Agar (MSA) to determine the abundance of *Staphylococcus* bacteria;
- *Pseudomonas* CN Agar to determine the abundance of bacteria of the genus *Pseudomonas* (PFA);
- Actinomycete Isolation Agar (AIA) to determine the abundance of *Actinomycetes*;
- Violet Red Bile with Glucose Agar (VRBG) to determine the abundance of bacteria of the genus *Enterobacteriaceae*.

Plates with VRBG and MSA media were incubated for 48 h at 37 °C, with TSBA and PFA media at 22 °C for 48 h, and with AIA and DRBC media at 22 °C for 7 days. After the incubation period, characteristic colonies were counted using a colony counter, and the results were expressed as cfu (colony-forming unit)∙g^−1^ of soil DW (dry weight) [40].

### 3.3. Ostracodtoxkit Test

The test determined the mortality and growth inhibition of *Heterocypris incongruens* after 6 days of exposure of these benthic ostracods to the test antibiotics at various concentrations. The tests were also carried out on a control sample containing no drugs. All the determinations were carried out in accordance with the recommendations of the procedure accompanying the test. The test was performed in accordance with ISO 14371:2012 [41].

### 3.4. Phytotoxicity Tests

The tests were conducted in accordance with the recommendations of the OECD/OCDE 208/2006 standard [42] and the PN-EN ISO 11269-2 standard [43]. The study determined the effect of AMP, AMX and their mixture (AMP:AMX = 1:1) applied at concentrations of 0.1, 1, 10, 50, 100 and 1000 mg∙kg^−1^ of soil dry weight (DW) on spring wheat (*Triticum aestivum* L.) of the Jutrzenka variety. The wheat was purchased from the Breeding and Production Plant in Nieznanice, which is part of the company Małopolska Plant Breeding—HBP Sp. z o.o. The tested antibiotics and their mixtures were weighed (to the nearest 0.0001 g) and dissolved in water. The test solution was then added to each batch of soil in the individual pots to achieve the desired concentration and mixed mechanically. Immediately after mixing, twenty seeds were sown into each vase containing 250 g of soil (loamy sand) with the addition of the tested antibiotics at specified concentrations or water (control). During the study, constant conditions were maintained in the vegetation hall at all times: temperature of 20 ± 2 °C, illumination at 170 µmol∙m^−2^∙s^−1^ under a 16 h day/8 h night regime and potting soil moisture of 70% ppw. The soil used for the study came from the company’s own field located in the village of Mokra, Silesia Province, Poland. The field is located more than 500 m from the road. The soil was subjected to detailed physicochemical and microbiological testing to confirm its applicability to the study. The soil used in the study was taken from the arable layer of the field. Basic soil parameters are checked in each sampled soil batch to confirm its compliance with the guidelines of OECD/OECD 206/2008.

All determinations were made in 4 replicates. Fourteen days after sowing the wheat seeds in the soil, the appearance of the tested plants was documented in digital photographs and the material was collected for analysis.

To determine the phytotoxicity of the tested antibiotics to wheat, in addition to visual assessment of changes in plant appearance, the seed germination potential (GP) and seed germination rate (GR) [44], the length of aboveground plant parts and their roots [45], dry weight content [46] and fresh weight yield of plants were determined. Due to the small effect of AMP, AMX and their mixture on plant growth, it was not possible to determine EC_50_ values.

### 3.5. Statistical Analysis

The data obtained were analyzed using STATISTICA 13.3, with one-factor ANOVA analysis of variance followed by Tukey’s post hoc test. Significant differences were considered at *p* < 0.05. All analyses were performed four times (n = 4), and results are presented as arithmetic mean ± standard deviation.

## 4. Conclusions

This study clearly shows that the tested antibiotics and their mixture have an effect on the number and composition of microorganisms living in the soil. As a result of the study, it was found that the presence of AMP and AMX and their mixture did not result in a reduction in the total abundance of microorganisms compared to the control objects, and even in the case of the mixture of these drugs, an increase in the total abundance of both bacteria and fungi was observed, regardless of their dosage. On the other hand, no negative response of ostracods to the presence of antibiotics in their habitat was observed.

The antibiotics used in the experiment also had little effect on the growth and development of the early stages of wheat. However, as the study only looked at the emergence and early development stages, it cannot be ruled out that exposure to the antibiotics tested could have an adverse effect on plants.

Negative effects on the test organisms were only observed at very high concentrations of antibiotics, which are not found in the environment. This means that, for the time being, amoxicillin, ampicillin and their mixtures should not have toxic effects on these environmental elements. However, we cannot rule out the possibility that these drugs may affect other organisms or interact with other drugs or compounds in the environment.

Despite the many studies that have been carried out in this area, it has still not been possible to clarify the effect of antibiotics on the environment. Many reports in the literature and our own research indicate that the effect of a particular drug, including antibiotics, on the environment depends on the type of drug, the concentration used, the organism affected and the duration of the effect. As the number of drugs used, and thus the number of drugs entering the environment, increases, it is necessary to continue detailed research to gain an in-depth understanding of the effects of drugs on various elements of the environment, including plants, which are important for the health and life of animals and humans.

## Figures and Tables

**Table 1:** Effect of AMP, AMX and AMP + AMX on *H. incongruens*.

Concentration of Antibiotics [mg·kg^−1^ of Soil DW]	AMP			AMX			AMP + AMX		
	Medium Length [µm]	Growth Inhibition [%]	Mortality [%]	Medium Length [µm]	Growth Inhibition [%]	Mortality [%]	Medium Length [µm]	Growth Inhibition [%]	Mortality [%]
Reference soil	980.56 ^ab^	-	0	980.56 ^ab^	-	0	980.56 ^ab^	-	0
0.1	984.88 ^ab^	5.03	0	982.19 ^ab^	5.40	0	973.69 ^a^	6.58	0
1	983.88 ^ab^	5.17	0	978.84 ^ab^	5.87	0	972.37 ^a^	6.77	0
10	985.70 ^ab^	4.91	0	987.40 ^ab^	4.68	0	970.55 ^a^	7.02	0
50	980.85 ^ab^	5.59	0	1025.95 ^a^	−0.68	0	986.53 ^a^	4.80	0
100	947.60 ^ab^	10.20	0	984.89 ^ab^	5.03	0	1000.94 ^a^	2.80	0
1000	917.13 ^b^	14.44	0	924.34 ^b^	13.44	0	933.51 ^a^	12.16	0

Data are means from 4 independent experiments. Values denoted by the same letters in the columns do not differ statistically at *p* < 0.05.

**Table 2:** Effect of ILs on the germination potential (GP) and germination rate (GR) of wheat. Data are means ± SD from 4 independent experiments. Values denoted by the same letters in the columns do not differ statistically at *p* < 0.05.

Concentration of Antibiotics (mg‧kg^−1^ of Soil DW)	AMP		AMX		AMP + AMX	
	GP (%)	GR (%)	GP (%)	GR (%)	GP (%)	GR (%)
0	88.3 ± 2.9 ^b^	95.0 ± 0.0 ^ab^	95.0 ± 8.7 ^a^	95.0 ± 8.7 ^a^	96.7 ± 5.8 ^a^	98.3 ± 2.9 ^a^
0.1	95.0 ± 5.0 ^ab^	96.7 ± 5.8 ^ab^	95.0 ± 5.0 ^a^	95.0 ± 5.0 ^a^	93.3 ± 5.8 ^a^	93.3 ± 7.6 ^a^
1	100.0 ± 0.0 ^a^	100.0 ± 0.0 ^a^	98.3 ± 2.9 ^a^	98.3 ± 2.9 ^a^	93.3 ± 5.8 ^a^	93.3 ± 5.8 ^a^
10	95.0 ± 0.0 ^ab^	96.7 ± 2.9 ^ab^	95.0 ± 5.0 ^a^	96.7 ± 5.8 ^a^	91.7 ± 2.9 ^a^	93.3 ± 2.9 ^a^
50	90.0 ± 5.0 ^ab^	90.0 ± 5.00 ^b^	98.3 ± 2.9 ^a^	98.3 ± 2.9 ^a^	91.7 ± 2.9 ^a^	95.0 ± 0.0 ^a^
100	95.0 ± 5.0 ^ab^	96.7 ± 2.9 ^ab^	96.7 ± 2.9 ^a^	98.3 ± 2.9 ^a^	96.7 ± 2.9 ^a^	98.3 ± 2.9 ^a^
1000	98.3 ± 2.9 ^ab^	98.3 ± 2.9 ^ab^	98.3 ± 2.9 ^a^	100.0 ± 0.0 ^a^	90.0 ± 5.0 ^a^	96.7 ± 5.8 ^a^

**Table 3:** Length of aboveground plant parts and roots, plant fresh weight yield, and dry weight content in wheat seedlings growing on soil containing AMP, AMX or a mixture of AMP + AMX. Data are means ± SD from 4 independent experiments. Values denoted by the same letters for the same biomarkers do not differ statistically at *p* < 0.05.

Concentration of Antibiotics (mg‧kg^−1^ of soil DW)	AMP	AMX	AMP + AMX
**Yield [g pot^−1^]**			
0	2.507 ± 0.104 ^c^	2.951 ± 0.594 ^a^	2.492 ± 0.146 ^a^
0.1	2.543 ± 0.084 ^c^	3.125 ± 0.132 ^a^	2.440 ± 0.258 ^a^
1	2.924 ± 0.157 ^ab^	3.207 ± 0.228 ^a^	2.495 ± 0.237 ^a^
10	2.729 ± 0.012 ^bc^	3.197 ± 0.121 ^a^	2.654 ± 0.084 ^a^
50	2.736 ± 0.072 ^bc^	3.142 ± 0.231 ^a^	2.599 ± 0.196 ^a^
100	2.943 ± 0.144 ^ab^	3.080 ± 0.123 ^a^	2.554 ± 0.007 ^a^
1000	3.192 ± 0.233 ^a^	3.086 ± 0.126 ^a^	2.520 ± 0.117 ^a^
**Dry weight [g g^−1^ FW]**			
0	0.0936 ± 0.0007 ^a^	0.0956 ± 0.0006 ^b^	0.0943 ± 0.0010 ^a^
0.1	0.0941 ± 0.0031 ^a^	0.0925 ± 0.0006 ^d^	0.0931 ± 0.0028 ^a^
1	0.0926 ± 0.0011 ^ab^	0.0929 ± 0.0005 ^cd^	0.0945 ± 0.0005 ^a^
10	0.0940 ± 0.0025 ^a^	0.0930 ± 0.0006 ^cd^	0.0925 ± 0.0009 ^a^
50	0.0902 ± 0.0014 ^ab^	0.0948 ± 0.0005 ^bc^	0.0942 ± 0.0004 ^a^
100	0.0914 ± 0.0019 ^ab^	0.0944 ± 0.0010 ^bcd^	0.0959 ± 0.0003 ^a^
1000	0.0882 ± 0.0011 ^b^	0.0989 ± 0.0011 ^a^	0.0947 ± 0.0010 ^a^
**Shoot length [cm]**			
0	14.3 ± 1.1 ^d^	18.6 ± 1.0 ^a^	15.5 ± 1.0 ^b^
0.1	15.0 ± 0.6 ^cd^	17.7 ± 0.4 ^a^	15.7 ± 0.7 ^ab^
1	15.9 ± 0.8 ^ab^	18.0 ± 0.7 ^a^	15.9 ± 0.9 ^ab^
10	15.7 ± 0.9 ^bc^	17.4 ± 3.2 ^a^	16.4 ± 1.0 ^a^
50	16.1 ± 0.9 ^ab^	17.7 ± 2.6 ^a^	16.2 ± 0.6 ^ab^
100	15.8 ± 0.8 ^abc^	18.0 ± 0.8 ^a^	15.7 ± 0.7 ^ab^
1000	16.7± 0.8 ^a^	18.4 ± 0.6 ^a^	15.9 ± 0.6 ^ab^
**Root length [cm]**			
0	13.1 ± 1.8 ^ab^	12.9 ± 1.2 ^a^	12.6 ± 1.6 ^ab^
0.1	13.0 ± 1.2 ^ab^	14.0 ± 1.1 ^a^	12.2 ± 0.8 ^b^
1	13.9 ± 1.1 ^a^	13.7 ± 1.2 ^a^	13.7 ± 1.0 ^a^
10	13.6 ± 1.1 ^ab^	14.1 ± 1.1 ^a^	13.8 ± 1.2 ^a^
50	14.0 ± 1.2 ^bc^	13.0 ± 1.0 ^a^	13.1 ± 1.0 ^ab^
100	12.3 ± 1.2 ^bc^	14.2 ± 1.3 ^a^	13.3 ± 1.2 ^ab^
1000	11.4 ± 0.9 ^c^	11.1 ± 1.3 ^b^	12.4 ± 0.9 ^b^

## Data Availability

The datasets used during the current research are available from the corresponding author on reasonable request.
